# Changes in Connexin-43 Expression and Neuropil Ultrastructure in the Cerebral Cortex of Bats During the Sleep–Wake Cycle

**DOI:** 10.3390/ijms27209171

**Published:** 2026-10-16

**Authors:** Stanislav Rodkin, Stanislav Bachurin, Rozaliia Nabiullina, Sergei Shebeko, Alexander Logvinov, Chizaram Nwosu, Evgeniya Kirichenko

**Affiliations:** 1 Research Laboratory “Medical Digital Images Based on the Basic Model”, Department of Bioengineering, Institute of Living Systems, Don State Technical University, Rostov-on-Don 344000, Russia; 2 Research Laboratory of Pharmacology and Experimental Pathology, Institute of Living Systems, Don State Technical University, Rostov-on-Don 344000, Russia

**Keywords:** connexin-43, gap junctions, hemichannels, sleep, wakefulness, molecular dynamics, neuropil ultrastructure, astrocytes

## Abstract

Sleep maintains central nervous system homeostasis, but many molecular mechanisms of sleep–wake regulation remain poorly understood. Connexin-43 (Cx43), the astrocytic gap junction protein, supports neuroglial communication, yet its role in physiological sleep is little studied. Here, we analyzed Cx43 expression, gap junction organization and neuropil ultrastructure in the motor cortex of common noctule bats (*Nyctalus noctula*) during sleep and wakefulness using confocal microscopy, transmission electron microscopy, semi-automated image analysis, and molecular dynamics. Sleep was associated with higher Cx43 immunoreactivity and with more and larger astrocytic gap-junction plaques than wakefulness. Across neuropil fields, synaptic, process and mitochondrial counts and total active-zone length were higher during wakefulness, and mean mitochondrial profiles were larger during sleep; after averaging within animals, only total active-zone length remained significantly greater during wakefulness. More perisynaptic astrocytic process (PAP) profiles contacted synapses during wakefulness, whereas individual PAPs were larger during sleep; both contrasts remained significant after animal-level averaging. As a secondary, hypothesis-generating analysis, we simulated histidine protonation of the Cx43 hemichannel as a simplified model of acidic conditions. Cerebral pH was not measured, and the simulations do not explain the sleep-associated Cx43 increase. In silico, protonation reduced conformational flexibility and narrowed the central and extracellular pore regions.

## 1. Introduction

Sleep is one of the fundamental physiological processes required for the restoration and maintenance of normal central nervous system function following periods of wakefulness [1]. Despite substantial progress in sleep research, many of the molecular and cellular mechanisms underlying this fundamental physiological process remain poorly understood. Astrocytes and their gap junctions play a pivotal role in maintaining neuroglial homeostasis during sleep. The principal structural component of astrocytic gap junctions is connexin-43 (Cx43), a 43 kDa transmembrane protein composed of four transmembrane domains, two extracellular loops, and cytoplasmic N- and C-terminal regions. Cx43 is essential for the formation of the astrocytic syncytium, which mediates spatial potassium buffering, intercellular transport of metabolites, and numerous other homeostatic processes [2,3]. Alterations in Cx43 expression or localization may substantially affect neuroglial homeostasis and consequently influence the sleep–wake cycle. Indeed, several studies have demonstrated that dysregulation of Cx43 expression leads to disturbances in sleep architecture and wakefulness [4,5]. Nevertheless, the physiological role of Cx43 in sleep regulation remains incompletely understood.

Synaptic activity is another key determinant of sleep regulation. According to the synaptic homeostasis hypothesis, synaptic strength progressively increases during wakefulness as a consequence of continuous interaction with the environment, whereas sleep promotes synaptic renormalization through reductions in synaptic strength, synaptic size, and/or synaptic density, thereby preventing synaptic saturation [6]. This concept has been supported by numerous experimental studies demonstrating state-dependent changes in synaptic density, axo-spinous synapse morphology, and active zone size during sleep and wakefulness [7]. However, these findings have been obtained almost exclusively in conventional laboratory rodents, primarily mice and rats.

In this context, bats represent an especially intriguing experimental model because of their unique physiological adaptations, including their characteristic upside-down sleeping posture, echolocation, exceptionally high metabolic rate [8], and prolonged daily sleep duration [9]. Together, these characteristics require a highly specialized central nervous system with remarkable neuronal plasticity, making bats an attractive model for investigating the cellular and molecular mechanisms underlying the sleep–wake cycle. Nevertheless, quantitative morphometric studies of cortical neuropil ultrastructure in bats under different physiological states remain virtually absent. In particular, no studies have comprehensively examined both Cx43 expression and ultrastructural parameters of the neuropil in sleeping and awake bats.

In our previous studies, we investigated Cx43 expression following traumatic brain injury in mice [10,11] and performed a systematic analysis of the role of Cx43 in neurological disorders associated with central nervous system pathology [12]. Those studies, in which ischemia and tissue acidosis often accompany injury, prompted us to examine the structural behavior of Cx43 under acidic conditions in silico. In the present work, this analysis is exploratory only. The inverted roosting posture of bats is a possible physiological setting in which acid–base shifts might occur; it is not evidence that cerebral acidosis develops during sleep. No pH or lactate measurements were obtained here.

The primary aim was to compare Cx43 expression and cortical neuropil ultrastructure between wakefulness and sleep in *Nyctalus noctula*. A secondary aim was to describe conformational effects of selected histidine protonation events on the Cx43 hemichannel and to discuss their limited, unverified relevance to the experimental findings. Because the design is cross-sectional, the results are interpreted as state-associated differences rather than as proof of a causal mechanism of sleep regulation.

## 2. Results

### 2.1. Three-Dimensional Reconstruction and Quantitative Analysis of Cx43 Expression by Confocal Microscopy

Confocal laser scanning microscopy was used to evaluate Cx43 expression in the motor cortex of bats during wakefulness and sleep (Figure 1). Representative confocal images showed that cell nuclei stained with SYTOX^TM^ Green exhibited normal morphology and were evenly distributed throughout the analyzed cortical region in both physiological states (Figure 1a). Immunolabeling for GFAP was used to confirm the astrocytic context of the Cx43 signal (Figure 1a). Three-dimensional reconstructions generated from confocal Z-stacks further revealed the spatial distribution of Cx43 and its colocalization with GFAP (Figure 1a).

The fluorescence intensity of Cx43 differed noticeably between physiological states. Sleep was characterized by stronger Cx43 immunoreactivity than wakefulness, as observed both in the three-dimensional reconstructions and in representative optical sections extracted from the confocal Z-stacks (Figure 1a,b).

Quantitative analysis of Cx43 fluorescence intensity confirmed the presence of statistically significant differences between the two physiological states (Figure 1c). Mean Cx43 fluorescence intensity was approximately 2.5-fold higher during sleep than during wakefulness (median 0.76 [0.50–1.80] vs. 0.30 [0.20–0.66], *p* < 0.05). A relatively broad range of individual values was observed in both groups, likely reflecting heterogeneous Cx43 distribution within the analyzed cortical region as well as interindividual variability among animals. Nevertheless, the difference between wakefulness and sleep remained statistically significant. Collectively, these findings show higher Cx43 immunoreactivity in bat motor cortex during sleep.

### 2.2. Morphometric Analysis of Electron Microscopy Images

Transmission electron microscopy combined with quantitative morphometric analysis revealed distinct differences in the neuropil ultrastructure of the bat motor cortex between wakefulness and sleep (Figure 2a). Overall, wakefulness was characterized by higher field-level values for most parameters associated with synaptic density and the cellular energy apparatus, whereas the mean size of active zones and neuronal processes did not differ.

The number of synapses was 50% higher during wakefulness than during sleep (Figure 2b; median 6 [5–7] vs. 4 [3–4.75], *p* < 0.001). Likewise, the number of synaptic zones increased by 69% during wakefulness (Figure 2c; 9.3 ± 2.92 vs. 5.5 ± 2.07, *p* < 0.001), whereas the total active zone length increased by approximately 1.7-fold (Figure 2d; median 2.32 [1.67–2.65] vs. 1.36 [1.12–1.70], *p* < 0.001). In contrast, the mean active zone length did not differ significantly between physiological states (Figure 2e; median 0.223 [0.209–0.268] vs. 0.259 [0.224–0.283], *p* = 0.194). These field-level contrasts were in the same direction, but only total active-zone length remained significant after animal-level correction.

A similar pattern was observed for neuronal processes. Wakefulness was associated with a 55% increase in the number of neuronal processes compared with sleep (Figure 2f; 12.35 ± 3.45 vs. 7.95 ± 2.19, *p* < 0.001), while the total neuronal process area increased by approximately 1.6-fold (Figure 2g; median 5.06 [3.99–6.78] vs. 3.18 [2.79–3.82], *p* < 0.001). However, the mean neuronal process area did not differ significantly between the two physiological states (Figure 2h; 0.439 ± 0.116 vs. 0.410 ± 0.123, *p* = 0.452).

Mitochondrial organization also differed between wakefulness and sleep. The number of mitochondria was 22% higher during wakefulness (Figure 2i; median 11 [10.25–13] vs. 9 [7.25–9], *p* < 0.001). Conversely, the mean mitochondrial area was 23% greater during sleep (Figure 2k; 0.232 ± 0.048 vs. 0.188 ± 0.048, *p* < 0.001), whereas the total mitochondrial area did not differ significantly between physiological states (Figure 2j; median 2.13 [1.50–2.81] vs. 1.82 [1.67–2.35], *p* = 0.499).

The same neuropil endpoints were averaged within each animal and compared again (Table 1). The animal-level results followed the field-level direction. After Holm correction, only total active-zone length remained significantly higher during wakefulness. Synapse number, synaptic-zone number, neuronal-process number and area, and mitochondrial number were also higher during wakefulness but did not meet the corrected threshold. Mean active-zone length and mean process area did not differ at either level. Mean mitochondrial area was greater during sleep across fields (Figure 2k) but did not remain different after averaging within animals; total mitochondrial area did not differ at either level.

Given the differences identified in the ultrastructural organization of the neuropil, an additional analysis of perisynaptic astrocytic processes (PAPs) was performed, revealing marked differences in their quantitative and morphometric characteristics between wakefulness and sleep (Figure 3). The number of PAP profiles contacting synapses was significantly higher during wakefulness than during sleep-median 3 (2.25–4.00) versus median 1 (1.00–2.00), respectively; *p* < 0.001 (Figure 3a). In contrast, mean PAP area was significantly greater during sleep—median 0.282 (0.175–0.367) µm^2^ versus median 0.137 (0.103–0.158) µm^2^ during wakefulness; *p* < 0.001 (Figure 3b). Total PAP area did not differ significantly between physiological states—median 0.418 (0.310–0.544) µm^2^ during wakefulness versus median 0.365 (0.205–0.548) µm^2^ during sleep; *p* = 0.626 (Figure 3c), indicating no statistically significant difference in the total PAP area within the analyzed ultrastructural fields. A similar pattern was observed for the perimeter of individual PAPs. Mean PAP perimeter was significantly greater during sleep—median 2.978 (2.482–3.539) µm versus median 1.910 (1.673–2.166) µm during wakefulness; *p* < 0.001 (Figure 3d). In contrast, total PAP perimeter did not differ significantly between physiological states-6.150 ± 2.374 µm during wakefulness versus 4.587 ± 2.653 µm during sleep; *p* = 0.057 (Figure 3e). Despite the numerically greater total PAP perimeter during wakefulness, this difference did not reach statistical significance.

The same endpoints were averaged within each animal and compared as a separate family (Table 2). The animal-level results followed the image-level pattern. After Holm correction, wakefulness was associated with a greater number of synapses contacted by PAPs, whereas sleep was associated with a larger mean PAP area and mean PAP perimeter. Total PAP area and total PAP perimeter did not differ between states, indicating a change in the size of individual processes rather than in their overall coverage of the neuropil field.

### 2.3. Model Detection Performance Relative to Human Correction

Cx43 detection counts and bounding-box areas were compared between two computational sources: raw model inference and human-corrected detections.

Object detection was performed using DINO [13] with a Swin-L backbone [14], as implemented in AutoGluon MultiModal v1.5.0 [15], which builds on MMDetection v3.3.0 [16] and MMEngine v0.10.7. All experiments were conducted in Python 3.10.12 with PyTorch 2.7.0 (CUDA 12.8, cuDNN 9.7.1) on a single NVIDIA GeForce RTX 4090 GPU (24 GB) running Ubuntu 22.04.4 LTS. AutoGluon was used for automated hyperparameter optimization on the PULLAR labeling and training platform. The dataset comprised 632 confocal microscopy images derived from 12 Z-stacks and was split into training and validation sets at an 80:20 ratio. These 12 Z-stacks were used only for model training and validation. Physiological-state comparisons used 13 independent animals after filtering (wakefulness, *n* = 6; sleep, *n* = 7). The split was performed at the Z-stack level to prevent data leakage between adjacent optical sections. Manual correction of model predictions was performed by an annotator blinded to physiological group [17]. Figure 4a summarizes the resulting detection and comparison pipeline. Representative confocal Z-stacks were processed by the DINO–SwinL model on the PULLAR platform to generate raw model inference. A subset of these detections was then independently reviewed and corrected by the blinded annotator, producing the human-corrected reference set. Direct comparison of the two sources on the same images identified detections retained by both and raw-only detections removed during manual correction. A second comparison examined these same metrics across the awake and sleep physiological states (representative images, Figure 4b–e; quantitative analysis, Figure 4f–i). The human-corrected detections were used as the reference for the state comparison, in order to avoid confounding the choice of detection source with the biological condition of interest.

Using the human-corrected detections as the reference, gap junction density was substantially higher during sleep than during wakefulness (Figure 4 f–i). Sleep images contained more than twice as many detected gap junctions per image (Figure 4f) as awake images (mean 280.8 vs. 129.4; mean difference −151.31, 95% CI [−157.99, −144.58]; Mann-Whitney U test, *p* < 0.001; Cohen’s d = 3.68, large effect). The total number of detected gap junctions per Z-stack was also significantly higher during sleep (Figure 4g). Gap junctions detected during sleep were also significantly larger than those detected during wakefulness (Figure 4h) (mean area 252.1 px^2^ vs. 192.8 px^2^; mean difference −59.26 px^2^, 95% CI [−65.35, −53.10]; *p* < 0.001; d = 1.59, large effect) and total detection area per Z-stack (Figure 4i). Both comparisons remained significant after Holm correction across all four statistical tests performed in this analysis.

The raw model detected more gap junctions per image than the human-corrected reference across all 656 paired images (mean 210.9 vs. 179.4 detections per image; mean difference +31.53, 95% CI [29.83, 33.21]; Wilcoxon signed-rank test, *p* < 0.001 after Holm correction; Cohen’s d = 1.40, large effect). Conversely, the bounding boxes retained after human correction were on average larger than those produced by the raw model (260.9 px^2^ vs. 229.7 px^2^; mean difference −31.21 px^2^, 95% CI [−34.31, −28.54]; *p* < 0.001; Cohen’s d = −0.83, large effect).

Pooling across all 656 images, the raw model achieved a precision of 0.798, recall of 0.939, F1 of 0.863, and a mean matched IoU of 0.845, indicating that the model recovered the large majority of human-identified gap junctions but also produced a non-trivial number of detections not retained after correction. Performance differed between physiological states (Figure 5a–d). Precision was lower for awake images than for sleep images (Figure 5a, 0.711 vs. 0.830), while recall was higher for awake images than for sleep images (Figure 5b, 0.976 vs. 0.928); F1 scores were correspondingly lower for awake than for sleep images (Figure 5c; 0.823 vs. 0.876), while mean matched IoU was comparable between conditions (Figure 5d, 0.841 vs. 0.849). At the subfolder level, precision ranged from 0.665 (awake_3) to 0.871 (sleep_2), recall ranged from 0.883 (sleep_2) to 0.996 (awake_6), and F1 ranged from 0.773 (awake_3) to 0.880 (sleep_1), with mean matched IoU remaining stable across subfolders (0.831–0.863).

### 2.4. Molecular Dynamics Analysis of the Cx43 Hemichannel

To examine how selected histidine protonation events affect Cx43 hemichannel geometry, MD simulations were performed for a physiological (reference) hexamer and a protonated hexamer. This analysis is hypothesis-generating and independent of the immunofluorescence and ultrastructural datasets. It does not explain the sleep-associated increase in Cx43 expression, and no cerebral pH was measured in the experimental animals.

Comparison of the final structures obtained after 100 ns of MD simulation revealed pronounced conformational differences between the physiological and protonated Cx43 hemichannels (Figure 6a). The RMSD between the final geometries, excluding hydrogen atoms, was 2.803 Å.

The spatial organization of the hemichannel pore also differed between the physiological and protonated forms (Figure 6b,c). Comparison of the two models demonstrated that protonation was accompanied by marked alterations in the geometry of the pore lumen.

Analysis of the RMSD trajectories demonstrated that the physiological hemichannel underwent greater conformational rearrangements than the protonated form (Figure 6d). This was reflected by higher initial RMSD values and a progressive increase in structural deviation from the starting conformation throughout the simulation.

Analysis of the radius of gyration further indicated that the physiological hemichannel maintained a less compact structure than the protonated form throughout the simulation (Figure 6e), suggesting greater conformational flexibility under physiological conditions.

The RMSF analysis identified two regions with the highest structural mobility, corresponding to amino acid residues 118–122 and 273–300 (Figure 6f), indicating increased flexibility of these domains.

Analysis of the pore-radius profile demonstrated that protonation reduced the diameter of both the central and extracellular regions of the pore while simultaneously increasing the pore diameter on the cytoplasmic side (Figure 6g). The same structural alterations were evident from comparison of the three-dimensional pore models (Figure 6b,c). These in silico findings suggest that the protonation scheme used here may restrict ion permeation through the Cx43 hemichannel under acidic conditions. They do not demonstrate ischemia or acidosis during bat sleep and do not account for state-dependent changes in Cx43 immunoreactivity.

## 3. Discussion

The present study describes state-associated differences in Cx43 immunoreactivity and cortical neuropil ultrastructure in bats, together with a separate computational analysis of a protonated Cx43 hemichannel. The data show higher Cx43 signal and larger gap-junction plaques during sleep, and higher synaptic and mitochondrial counts during wakefulness. They do not establish that increased Cx43 expression is a causal mechanism of neuroglial homeostasis.

Confocal laser scanning microscopy combined with three-dimensional reconstruction of confocal Z-stacks revealed a significant increase in Cx43 immunoreactivity during sleep compared with wakefulness. These findings are consistent with previous studies demonstrating that Cx43 plays a pivotal role in regulating the sleep–wake cycle by mediating metabolic coupling between astrocytes and neurons. Astrocyte-specific deletion of Cx43 in mice results in excessive sleepiness and impaired maintenance of wakefulness during the active phase [4]. Furthermore, sleep deprivation followed by recovery sleep in rats has been shown to produce opposite changes in Cx43 and Cx36 expression, with increased Cx43 accompanied by decreased Cx36 levels in the hypothalamus [5]. Cx43 expression within the suprachiasmatic nucleus has also been reported to play an essential role in maintaining circadian rhythm stability under constant darkness and in facilitating circadian re-entrainment following alterations in the light–dark cycle [18]. Therefore, the elevated Cx43 immunoreactivity observed during sleep is consistent with enhanced astrocytic coupling after wakefulness. Increased Cx43 could support spatial ion buffering, metabolic transfer, and glymphatic clearance [19], but this was not tested here. Time of day, prior activity, capture stress, and behavioral state assignment remain possible confounders. All histology was obtained from motor cortex; neighboring sensory fields were not sampled. Recent use of this region could therefore contribute to the synaptic and Cx43 contrasts and cannot be fully separated from sleep–wake state.

In addition, semi-automated analysis of confocal Z-stacks demonstrated that both the density and size of Cx43-positive gap junction plaques were significantly greater during sleep than during wakefulness. Based on the human-corrected detections, sleep images contained more than twice as many gap junction plaques, which also exhibited significantly larger plaque areas than those detected in wakefulness. These differences remained statistically significant after correction for multiple comparisons and were associated with large effect sizes.

Quantitative analysis of Cx43 gap junction plaques under conditions of variable image quality is, however, associated with several methodological challenges. The human-in-the-loop workflow employed in the present study addresses a long-standing need in connexin imaging for automated, cell-resolved, and structure-resolved quantification of gap junction plaques. As previously noted, conventional approaches based on fluorescence density measurements lack both automation and spatial assignment of connexin signals to individual cells [20]. The bounding-box-based analysis pipeline developed in this study represents a further step toward automated plaque quantification. Rather than recovering plaques missed by the raw model, manual correction primarily removed spurious detections: precision of 0.798 indicates that roughly 20% of raw-model detections were not retained after correction, while recall of 0.939 indicates that only ~6% of human-identified plaques were missed by the raw model. The systematic enlargement of bounding boxes following manual correction, together with the lower precision observed in wakefulness compared with sleep, most likely reflects the nanoscale heterogeneity of gap junction architecture and the complex topography of the plasma membrane. Previous studies using super-resolution and volume electron microscopy have demonstrated that gap junction plaques possess intricate substructures that may be under- or over-segmented by conventional fluorescence-based detection algorithms depending on plaque density and local image quality [21]. Likewise, the higher recall accompanied by lower precision observed in wakefulness is consistent with the limitations reported for general-purpose spot-detection algorithms, which typically sacrifice specificity to maximize sensitivity under conditions of reduced signal-to-noise ratio. These observations support the interpretation that the discrepancies between automated and manually corrected detections in the present study were attributable, at least in part, to image quality-dependent detection complexity rather than to systematic algorithmic bias [22].

The ultrastructural findings are compatible with the synaptic homeostasis hypothesis. After averaging within animals and Holm correction, only total active-zone length remained significant; the other synaptic and process counts kept the same direction but did not meet the corrected threshold. In contrast, the mean size of individual active zones and neuronal processes did not differ significantly between physiological states. Interestingly, these ultrastructural changes exhibited an inverse relationship with Cx43 expression, which was markedly reduced during wakefulness. This pattern matches reports of higher synaptic content during wakefulness [6]. Because each bat was sampled only once, however, we cannot say whether synapses in the same animal become stronger in wake and weaker in sleep. Furthermore, ultrastructural studies in mice have shown that axo-spinous synaptic interfaces are enlarged during wakefulness relative to sleep [7], which is consistent with the same direction of effect.

Additional analysis of PAPs provided further insight into the organization of astrocytic processes around synaptic terminals as a function of physiological state. PAPs are a dynamic component of the synaptic environment and can alter their spatial interactions with synaptic structures depending on the level of neuronal activity [23]. In the present study, a greater number of PAP profiles contacting synapses was observed during wakefulness, whereas individual PAPs exhibited greater area and perimeter during sleep. The same pattern was present after averaging within animals. At the same time, total PAP area did not differ between the physiological states, indicating a remodeling of the morphometric organization of PAPs rather than a simple increase or decrease in their overall coverage.

Interestingly, the changes in PAPs occurred against an opposite pattern of Cx43 expression: Cx43 levels and the density of Cx43-positive gap junction plaques were increased during sleep, whereas the number of PAP profiles contacting synapses was reduced. This indicates that the increase in Cx43 expression cannot be explained simply by an increase in the number of PAPs. At the same time, Cx43 is localized in small astrocytic processes in close proximity to synaptic complexes [24], suggesting a potential relationship between Cx43 and PAPs. Thus, changes in Cx43 and PAPs may represent distinct, although potentially interconnected, components of astrocytic remodeling across the sleep–wake cycle.

Ultrastructural analysis also revealed state-dependent changes in mitochondrial organization. The animal-level tests are reported in Table 1; the section-level counts are not treated as a separate result. Wakefulness was characterized by an increased number of mitochondria accompanied by a reduction in their mean area, whereas sleep was associated with fewer but significantly larger mitochondria. These observations may reflect dynamic regulation of mitochondrial fission and fusion according to the functional state of the brain. Mitochondria, as the primary organelles responsible for ATP production and cellular metabolism, are tightly coupled to neuronal activity, and their structural dynamics constitute a highly adaptive mechanism that rapidly responds to neuronal energy demands [25]. During wakefulness, when synaptic activity is elevated, efficient distribution of energy resources is likely achieved through enhanced mitochondrial fission, resulting in an increased number of smaller mitochondria. Conversely, the reduced metabolic demands associated with sleep may favor mitochondrial fusion, leading to the formation of larger mitochondria, thereby promoting more efficient ATP production and facilitating mitochondrial maintenance and repair. Consistent with this interpretation, studies in *Drosophila* have demonstrated that mitochondrial fusion predominates in neurons during sleep, whereas wakefulness is associated with increased mitochondrial fragmentation in response to elevated energetic demands [26]. Furthermore, sleep deprivation has been reported to induce mitochondrial enlargement, the appearance of abnormal mitochondrial morphologies, and other ultrastructural alterations indicative of disrupted mitochondrial homeostasis [27]. Prolonged wakefulness has also been shown to increase mitochondrial DNA copy number and the expression of proteins involved in mitochondrial biogenesis and oxidative phosphorylation, providing additional evidence of mitochondrial activation under sustained wakeful conditions [28].

Given the sleep-associated increase in Cx43 immunoreactivity, we were also interested in the pH-dependent regulation of the Cx43 hemichannel, which is well-documented independently of protein expression. Bats roost upside down, and this posture could, in principle, alter cranial hemodynamics; whether it produces measurable cortical acidosis is unknown, and no pH or lactate data were collected here. On that limited rationale we performed a hypothesis-generating MD comparison of a reference and a protonated Cx43 hexamer, as a simplified model of acidic conditions, without treating the simulations as evidence that acidosis occurs during sleep or as an explanation of the Cx43 immunofluorescence results.

The final geometries of the physiological and protonated Cx43 hemichannels obtained after 100 ns of molecular dynamics simulation revealed pronounced conformational differences. The most prominent alterations involved the pore architecture, where protonation resulted in narrowing of the central and extracellular regions of the channel while simultaneously widening the cytoplasmic entrance. These findings are consistent with the established mechanism of pH-dependent regulation of Cx43 hemichannels. Previous studies have shown that lowering intracellular pH to approximately 6.5 enhances the interaction between the L2 domain and the C-terminal domain of Cx43 [29,30]. According to the “ball-and-chain” gating model, the C-terminal domain functions as a mobile gating element that binds to the L2 region under acidic conditions, thereby stabilizing the closed state of the channel [31]. Moreover, in our previous in silico study, we demonstrated that local acidosis (pH ≈ 6.5) promotes pathological structural rearrangements of Cx43. Specifically, acidic conditions strengthened interactions between the L2 and C-terminal domains and induced noncanonical residue interactions that may interfere with normal channel function [10].

Analysis of the dynamic parameters further demonstrated that the protonated hemichannel exhibited lower conformational flexibility than the physiological form, as reflected by reduced RMSD values throughout the simulation, a more compact overall structure, and stabilization of highly mobile regions corresponding to residues 118–122 and 273–300. Hemichannel permeability is known to be highly dynamic and can change within seconds to minutes in response to multiple regulatory factors, including intracellular pH [32]. In particular, connexin hemichannels directly sense cytoplasmic pH, and acidification induces rapid and reversible channel closure independently of calcium signaling and soluble intracellular mediators [33]. This mechanism is likely to be physiologically important, as selective inhibition of Cx43 hemichannels has been shown to exert pronounced neuroprotective effects, including reductions in infarct volume, cerebral edema, white matter damage, and cognitive impairment following ischemic injury [34]. Those observations concern pH gating and injury models; they do not show that hypoxia or acidosis occurred during sleep in the present animals, and they do not explain the sleep-associated increase in Cx43 immunoreactivity.

Taken together, the increase in number and size of Cx43-positive plaques during sleep is compatible with stronger coupling within the astrocytic syncytium and with greater capacity for ion buffering and metabolic support. Wakefulness, by contrast, was marked by a greater total active-zone length after animal-level averaging, with the other synaptic counts in the same direction, together with lower Cx43 immunoreactivity—a pattern consistent with elevated synaptic load and energy demand. The MD results indicate that histidine protonation can narrow the hemichannel pore and reduce conformational flexibility, in line with known pH-dependent gating of Cx43. That computational observation, however, remains separate from the histological findings: cerebral pH was not measured, and protonation does not account for the sleep-associated rise in Cx43 signal. Overall, the data describe coordinated, state-associated differences between wakefulness and sleep in bat motor cortex. They suggest a plausible shift from high synaptic demand to a more coupled, potentially restorative glial state, without establishing a causal mechanism of sleep regulation.

## 4. Materials and Methods

### 4.1. Ethical Approval

Adult male common noctule bats (*Nyctalus noctula*; *n* = 14) captured in the vicinity of Rostov-on-Don, Russia, were used to investigate Cx43 expression and neuropil ultrastructure during the sleep–wake cycle. These were wild-caught animals, not a laboratory-bred colony and not zoo specimens. Animals were housed in specialized aviaries designed to allow their natural upside-down roosting behavior, with ad libitum access to food and water. Housing conditions were maintained close to the natural environment, including an ambient temperature of 20–25 °C, relative humidity of 50–70%, effective ventilation (18 air exchanges per hour), and a controlled light–dark cycle with a predominance of the dark phase.

All experimental procedures were performed in accordance with international and national guidelines for the ethical use of wild animals in research. The study complied with Directive 2010/63/EU of the European Parliament and of the Council of 22 September 2010 on the protection of animals used for scientific purposes, as well as the relevant legislation of the Russian Federation, including the Federal Law “On Wildlife” (No. 52-FZ, 24 April 1995), the Rules of Good Laboratory Practice (Order No. 708n of the Ministry of Health of the Russian Federation, 23 August 2010), GOST 33215-2014 (Guidelines for Accommodation and Care of Laboratory Animals), and regulations governing the handling of wild mammals. Animal capture was performed under permits issued by the regional environmental authorities. The experimental protocol (No. 5) was approved by the Bioethics Committee of Don State Technical University on 15 March 2023.

### 4.2. Animals and Experimental Procedure

Adult male common noctule bats (*Nyctalus noctula*; *n* = 14 captured) were used. Animals were assigned to two experimental groups according to their physiological state: wakefulness and sleep. After exclusion of animals with technical failures during tissue processing, the final sample sizes differed by assay. For confocal Cx43 analyses with human-corrected detections, *n* = 6 wakefulness Z-stacks and *n* = 7 sleep Z-stacks were retained. For electron-microscopic morphometry, 20 neuropil fields per physiological state were obtained from 6 animals during wakefulness and 6 animals during sleep. The captured cohort of 14 animals therefore does not equal the number of datasets entering every analysis; exact n is reported with each result.

Physiological state was determined by behavioral observations. Sleeping animals were identified by their characteristic upside-down hanging posture, closed eyes, and markedly reduced locomotor activity. State assignment was therefore behavioral rather than electrophysiological, and each animal contributed a single terminal observation.

Animals were euthanized during the appropriate functional state: wakeful animals during their active period and sleeping animals during consolidated sleep while minimizing the likelihood of arousal. Following isoflurane anesthesia, animals were euthanized and tissues were fixed. For confocal microscopy, tissues were fixed in 4% paraformaldehyde (PFA) prepared in 0.1 M phosphate-buffered saline (PBS, pH 7.4), whereas samples intended for transmission electron microscopy and morphometric analysis were fixed in a mixture of 2.5% glutaraldehyde and 2% PFA prepared in the same buffer.

### 4.3. Confocal Laser Scanning Microscopy

To investigate Cx43 expression in the motor cortex of *Nyctalus noctula*, brains fixed in 4% PFA were sectioned coronally at a thickness of 20 μm using a Leica VT1000 S vibratome (Leica Biosystems, Nussloch, Germany). Adjacent sensory fields were not sampled. Sections were subsequently incubated in 15% and 30% sucrose solutions for 1 h each for cryoprotection and then stored at −80 °C.

Sections were washed with PBS and incubated for 1 h at room temperature in a blocking solution containing 5% bovine serum albumin (BSA; Sisco Research Laboratories Pvt. Ltd., Mumbai, India) and 0.3% Triton X-100 (Sisco Research Laboratories Pvt. Ltd., Mumbai, India) to minimize nonspecific antibody binding. The sections were then incubated for 48 h at 4 °C with rabbit anti-Cx43 primary antibody (1:100; E-AB-70097; Elabscience Biotechnology Inc., Houston, TX, USA) and mouse anti-GFAP primary antibody (1:1000; SAB4200571; Sigma-Aldrich, St. Louis, MO, USA).

After extensive washing with PBS, sections were incubated with goat anti-rabbit IgG H&L secondary antibody conjugated to Alexa Fluor^®^ 555 (AB150078; Abcam, Cambridge, UK) and goat anti-mouse IgG H&L secondary antibody conjugated to Alexa Fluor^®^ 647 (ab150115; Abcam, Cambridge, UK).

Negative controls were processed without primary antibodies. Cell nuclei were stained with SYTOX^TM^ Green Nucleic Acid Stain (Thermo Fisher Scientific, Waltham, MA, USA) diluted 1:1000 in PBS and incubated for 20–30 min at room temperature in the dark. Following staining, sections were washed three times with PBS to remove excess dye and reduce background fluorescence. Samples were mounted in an antifade mounting medium (Abberior GmbH, Göttingen, Germany) and coverslipped.

Images were acquired using an Abberior Facility Line inverted confocal laser scanning microscope (Abberior Instruments GmbH, Göttingen, Germany). Three-dimensional image stacks were obtained with a Z-step of 200 nm and a pixel size of 40 nm. Three-dimensional reconstructions were generated using ImageJ (version 1.54j, National Institutes of Health, Bethesda, MD, USA).

Quantitative analysis of Cx43 expression was performed in ImageJ. A rectangular region of interest encompassing the entire field of view was selected for each image, and the mean fluorescence intensity was measured. Background fluorescence was determined in an adjacent area lacking specific staining and subsequently subtracted from the measured fluorescence intensity for each image.

### 4.4. Transmission Electron Microscopy

To examine neuropil ultrastructure in the motor cortex of *Nyctalus noctula* during wakefulness and sleep, motor-cortex samples were immersion-fixed in 2.5% glutaraldehyde (Aurion, Eugene, OR, USA) and 2% paraformaldehyde in 0.1 M PBS (pH 7.4). After washing with PBS, specimens were post-fixed in 1% osmium tetroxide (OsO_4_) prepared in PBS for 1.5 h to stabilize membrane structures.

Samples were dehydrated through a graded ethanol series (50–100%) followed by three changes in propylene oxide and embedded in Epon-812 epoxy resin.

Semi-thin and ultrathin sections were prepared using an EM UC7 ultramicrotome (Leica Microsystems, Wetzlar, Germany) equipped with an Ultra 45° diamond knife (Diatome, Biel/Bienne, Switzerland). Ultrathin sections were contrasted with uranyl acetate and lead citrate before examination.

Ultrastructural analysis was performed using a JEM-1011 transmission electron microscope (JEOL, Akishima, Japan) operated at an accelerating voltage of 80 kV. Images were recorded at a primary magnification of ×40,000 with an Erlangshen ES500W CCD camera (Gatan, Pleasanton, CA, USA) using Digital Micrograph software (Gatan, Pleasanton, CA, USA). Scale bars were calibrated from the microscope magnification.

Morphometric analysis of electron micrographs was performed using ImageJ (version 1.51r). Twenty neuropil fields were analyzed for each physiological state. Fields were taken from layer IV neuropil of motor cortex; layer was identified from the position of the block in the coronal section. Each field was taken from a separate ultrathin section, from 6 animals during wakefulness and 6 during sleep. On average, about three ultrathin sections, and thus about three images, were obtained from each animal. Images from the same animal were treated as repeated measures, not as independent observations. The seventh sleep animal was excluded from electron microscopy because of a technical artifact and was retained only in the confocal analysis. The quantified parameters were the number of synapses, the number of neuronal processes, the mean and total neuronal-process area, the number of synaptic zones, the mean and total active-zone length, and the number, mean area, and total area of mitochondria. On the same fields, PAPs were identified. We additionally quantified the number of synapses contacted by at least one PAP, the mean and total PAP area, and the mean and total PAP perimeter. Group comparisons were first performed at the level of individual sections, without averaging within animals. The same endpoints were then averaged within each animal, and the group comparisons were repeated on these animal means.

### 4.5. Model Training and Ground Truth Annotation

Ground-truth annotations for model training and evaluation were generated manually using the PULLAR annotation platform [17]. A single trained annotator labeled all images to ensure consistent annotation criteria. The annotated image set was split at the level of individual Z-stacks rather than individual frames, so that no Z-stack contributed images to both the training and evaluation sets. Within each Z-stack, frames were sampled across the full imaging depth to capture the structural variability present at different optical sections, rather than annotating only a contiguous subset of slices.

The detection model was trained using AutoGluon’s multimodal framework (autogluon.multimodal, version 1.5), applying its highest-accuracy configuration preset, which automatically selects among a predefined set of competitive computer-vision architectures and training strategies without requiring manual architecture selection. The underlying detector combines a DINO-based (DETR with Improved deNoising anchOr boxes model as core model) object detection head with a Swin Transformer (Large) backbone, chosen for its strong feature representation capacity in dense object detection tasks such as the present application. Training proceeded for 100 epochs with a batch size of 4, as validation loss plateau, with random flipping and rotation augmentations applied. Hyperparameter selection was handled automatically by AutoGluon’s internal search procedure.

Confocal Z-stack images of Cx43-immunolabeled brain tissue were acquired from animals in two physiological states, each one as image set: wakefulness (6 image sets) and sleep (7 image sets). Each Z-stack image was processed by an automated deep-learning model trained on normal mouse brain tissue to identify candidate gap junctions, with each detection characterized by a bounding box (position, width, height). For each image, two parallel sets of detections were available: the raw, unmodified model inference, and the same detections after manual correction by a trained human operator, who reviewed and adjusted the model output. Three filtering steps were applied to both detection sources prior to statistical analysis, in the following order: (1) a hard area cap excluding individual detections with a bounding-box area exceeding 500 px^2^, a threshold set above the observed range of genuine gap junction sizes (~150–330 px^2^) and intended to remove rare, anatomically implausible detections arising from box-merging errors at tissue edges; (2) zero-tail exclusion, removing Z-stack planes in which all available detection sources reported zero detections, treated as out-of-tissue optical sections beyond the physical extent of the labeled tissue; and (3) image-level outlier exclusion, in which the mean detection area per image was computed for each image subset and images whose mean area exceeded 3 standard deviations from the subset mean were excluded, removing isolated images affected by likely segmentation or staining artifacts without requiring manual curation. After filtering, 656 images retained both raw and human-corrected detections and were used for the detection-source comparison, while 290 awake images and 366 sleep images with valid human-corrected detections were retained for the physiological-state comparison.

### 4.6. Molecular Dynamics Simulations

Molecular dynamics (MD) simulations were performed using GROMACS 2026.2 [35]. The simulated object was a Cx43 hemichannel hexamer, not a single subunit, embedded in a lipid bilayer. The initial structural model was obtained from the Protein Data Bank (PDB ID: 7F93) [36] and completed using the Boltz-2 package [37]. Missing disulfide bonds within the Cx43 molecule were restored between cysteine residues Cys54-Cys198, Cys61-Cys192, Cys65-Cys187, and Cys271-Cys298.

A lipid bilayer surrounding the connexin hemichannel (hexamer) was generated using Packmol–Memgen [38]. Two simulation systems were prepared: a reference model and a protonated model. The protonated system is a computational construct and is not based on pH measured in the experimental animals. To identify histidine residues predicted to become protonated under acidic conditions, PROPKA 3 [39] was employed. Only His142, His194, and His351 were modeled in the protonated (HIP) state; other titratable residues were left in their standard physiological protonation states. An explicit target pH was not imposed beyond this discrete protonation scheme.

Water boxes measuring 15 × 15 × 4 nm were constructed above and below the membrane plane. Water molecules were randomly replaced with K^+^ ions to neutralize the total system charge. All system preparation steps were performed using AmberTools26 [40].

The MD simulation protocol consisted of the following steps:

1. Energy minimization using the steepest descent algorithm;
2. Temperature equilibration to ensure uniform solvent distribution on both sides of the membrane;
3. Energy minimization of the equilibrated system using the L-BFGS algorithm;
4. Sequential temperature and pressure equilibration;
5. A 50 ns equilibration simulation to stabilize the system;
6. A 100 ns production MD simulation. A 100 ns production run is short relative to the slowest motions of a large oligomeric channel and should be interpreted as a comparative, hypothesis-generating snapshot rather than exhaustive sampling. Independent replica simulations were not performed.

Temperature equilibration and production simulations were performed using the semi-isotropic C-rescale barostat to account for the anisotropic nature of the membrane-containing system at 310 K and 1 atm. The following force fields were employed: ff19SB for proteins, Lipid21 for membrane lipids, and the OPC water model for water molecules and ions.

Trajectory analysis included calculation of the root-mean-square deviation (RMSD) of hemichannel atomic coordinates relative to the initial aligned structure, the radius of gyration (overall and along the pore axis, Z), root-mean-square fluctuations (RMSFs) in amino acid residues, and pore-radius profiles as a function of the channel coordinate. Pore geometry was analyzed using the HOLE-2 program [41]. RMSF values are reported as the average per residue of a single representative subunit after hexamer alignment; they are not a residue-wise average over all six subunits unless stated otherwise.

### 4.7. Statistical Analysis

Statistical analysis was performed in SigmaPlot (version 12.5, Systat Software, USA) and Python (version 3.13.7) using the SciPy (version 1.18.0), statsmodels, and Matplotlib (version 3.11.1) libraries. The experimental unit for confocal analyses was the animal (one Z-stack per animal). For electron microscopy, 20 neuropil fields were analyzed for each physiological state. Each field was taken from a separate ultrathin section obtained from one of six animals (on average, three sections per animal). Fields were sampled from the same motor-cortex region, using neuropil corresponding to layer IV. Layer was identified from the position of the block in the coronal section, not by a laminar marker on the ultrathin section itself. Measurements from the same animal were treated as repeated measures, not as independent observations. Because the number of animals was small, the same endpoints were evaluated at two levels. Section-level analysis, without averaging, was used to show whether the direction of each contrast was shared across the sampled fields rather than produced by a single animal (Figure 2 and Figure 3). Endpoints were then averaged within each animal and the comparisons were repeated, so that a bat contributing more readable sections did not weigh more than the others (Table 1 and Table 2). The two levels answer different questions and were not treated as two independent proofs. With only six animals per state, the animal-level tests are the conservative comparison; the section-level tests show how consistent that pattern was across the available sections. The seventh sleep animal was excluded from electron microscopy because of a technical artifact and was retained only in the confocal analysis.

Normality of distribution was assessed with the Shapiro–Wilk test, and homogeneity of variances with the Brown–Forsythe test. Because the assumptions of parametric tests were not met, two independent groups were compared with the two-sided Mann–Whitney U test. Raw model detections and human-corrected detections obtained from the same frames were compared with the Wilcoxon signed-rank test. Data are presented as median [Q1–Q3]. Effect size was quantified using the rank-biserial correlation coefficient. The rank-biserial correlation was reported for the electron-microscopic comparisons, after averaging within animals (Table 1 and Table 2). A positive value indicates a higher result during wakefulness, and a negative value a higher result during sleep. Cohen’s d was reported only for the image-level Cx43 detection contrasts, as a descriptive effect size, and was not used to decide significance.

Multiple comparisons were controlled with Holm’s sequentially rejective Bonferroni procedure within prespecified families of tests: neuropil ultrastructural endpoints; PAP endpoints; four Cx43 plaque metrics after manual correction; and four automatic-detection performance metrics (precision, recall, F1, and mean matched IoU). The section-level and animal-level electron-microscopy families were corrected separately. Mean Cx43 fluorescence intensity was analyzed as a single primary intensity contrast without multiplicity correction. Differences were considered statistically significant at two-sided *p* < 0.05 after the corresponding correction.

Bootstrap resampling was not used for hypothesis testing and was not applied to the electron-microscopic morphometry. Ninety-five percent confidence intervals for mean differences were obtained by bootstrap resampling: samples of the same size were drawn 10,000 times, the mean difference was calculated for each replicate, and the 2.5th and 97.5th percentiles were used as interval bounds; the random-number generator was fixed at seed 42. These intervals were calculated only for the Cx43 detection comparisons, after the Mann–Whitney and Wilcoxon tests, and at the image level (290 awake and 366 sleep frames for the state comparison; 656 paired frames for raw versus human-corrected detections). They are therefore narrower than an animal-level interval would be and were not used to decide significance. Between-group comparisons of physiological states used only human-corrected detections.

No a priori power calculation was performed; sample size was determined by animal availability, permit constraints, and the 3R principles. After exclusion of samples with technical artifacts, the confocal analysis included *n* = 6 animals during wakefulness and n = 7 animals during sleep. Electron microscopy included *n* = 6 animals in each state. Given the limited sample size, the results were interpreted as associations rather than as evidence of causal relationships. Manual correction of Cx43 detections was performed by an annotator blinded to physiological group.

## 5. Limitations of the Study

The present findings should be read in light of the study design. The number of animals was limited, and the comparison of sleep and wakefulness was cross-sectional: each bat was examined once, so the data describe state-associated differences rather than within-animal trajectories. State was assigned behaviorally, without EEG, and time of day, prior motor activity, and capture conditions may have contributed to the observed contrasts.

All histological material was obtained from motor cortex. This defines the anatomical scope of the work: the changes reported here refer to that region and are not intended as a description of the neocortex as a whole. GFAP was used to confirm the astrocytic context of the Cx43 signal and was not treated as a separate quantitative endpoint. PAPs were identified by ultrastructural criteria on single sections, and mitochondria were scored across the neuropil field; this describes tissue organization on the available material but does not partition neuronal and astrocytic compartments.

Molecular dynamics of the protonated Cx43 hemichannel was included as an additional in silico analysis of the channel’s sensitivity to protonation. Cerebral pH was not measured, and this block is not used to explain the immunohistochemical differences. Quantification of Cx43 plaques relied on human-corrected detections, which strengthens the between-group comparison while remaining dependent on the quality of the source images.

## Figures and Tables

**Figure 1:**
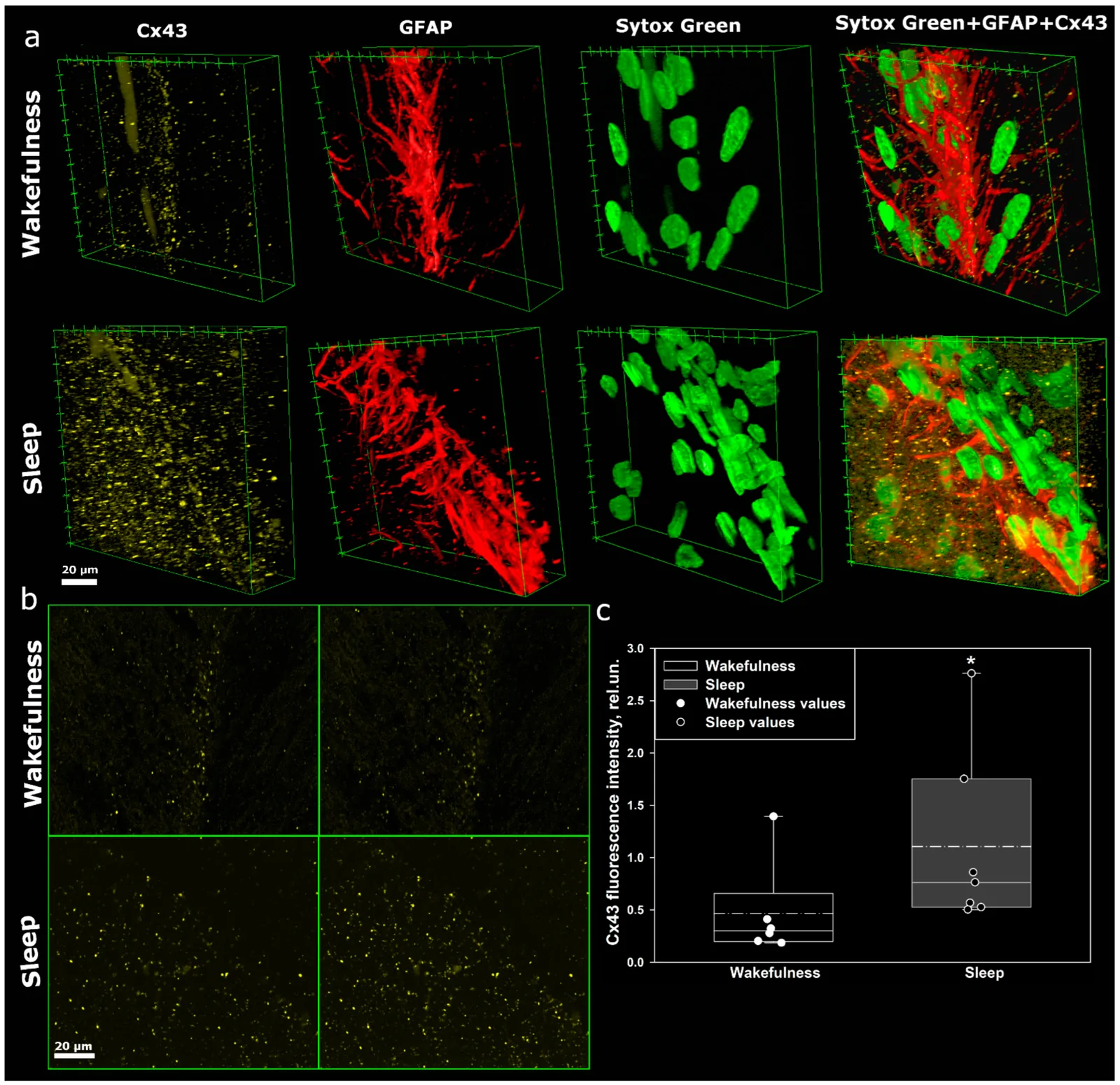
Cx43 expression in the cerebral cortex of bats during wakefulness and sleep. (**a**) Representative three-dimensional reconstructions generated from confocal Z-stacks obtained during wakefulness and sleep. Yellow fluorescence corresponds to Cx43 immunolabeling, red indicates the astrocytic marker GFAP, and green represents nuclear staining with SYTOX^TM^ Green. Marker colocalization is shown as merged images (SYTOX™ Green + GFAP + Cx43) with three-dimensional reconstruction. (**b**) Representative optical sections extracted from confocal Z-stacks showing Cx43 immunolabeling during wakefulness and sleep. Scale bar, 20 μm. (**c**) Quantitative analysis of mean Cx43 fluorescence intensity (wakefulness, *n* = 6 animals; sleep, *n* = 7 animals). Data are presented as box-and-whisker plots. The solid horizontal line indicates the median, the dashed line represents the mean, boxes denote the interquartile range (25th–75th percentile), and whiskers extend to the minimum and maximum values excluding outliers. Statistical analysis was performed using the two-sided Mann–Whitney U test. * *p* < 0.05 indicates statistically significant differences between wakefulness and sleep. Black circles represent wakefulness, and white circles represent sleep.

**Figure 2:**
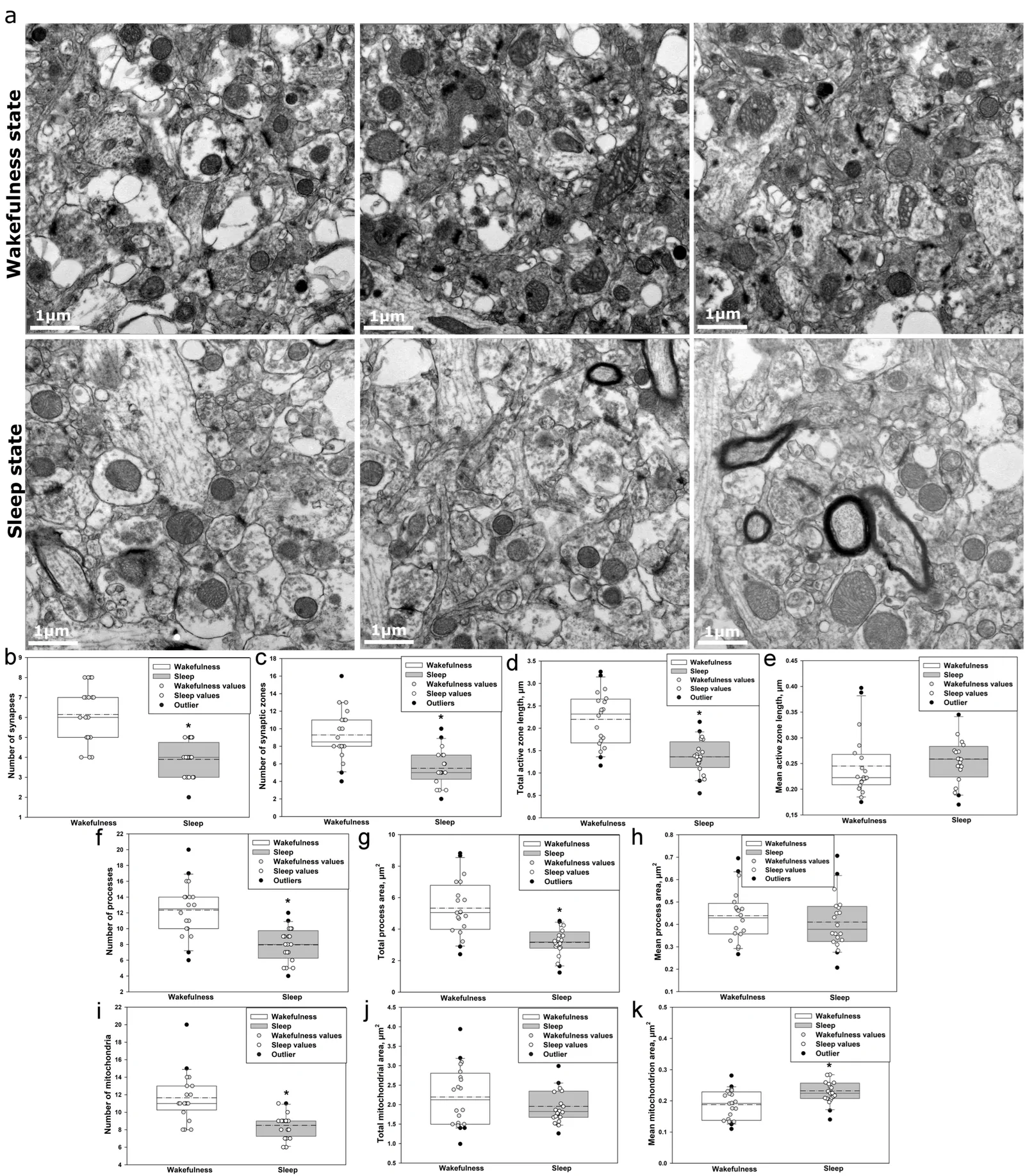
Ultrastructural characteristics of the motor cortex in bats during wakefulness and sleep. (**a**) Representative transmission electron micrographs of the neuropil during wakefulness and sleep. Scale bar, 1 μm. Synapses: (**b**) Number of synapses. (**c**) Number of synaptic zones. (**d**) Total active zone length. (**e**) Mean active zone length. Neuronal processes: (**f**) Number of neuronal processes. (**g**) Total neuronal process area. (**h**) Mean neuronal process area. Mitochondria: (**i**) Number of mitochondria. (**j**) Total mitochondrial area. (**k**) Mean mitochondrial area. Analyses included 20 neuropil fields per physiological state, obtained from 6 animals during wakefulness and 6 animals during sleep. Statistical analysis was performed using the two-sided Mann–Whitney U test with Holm’s sequentially rejective Bonferroni correction within this family of ultrastructural endpoints. * *p* < 0.05 after correction indicates statistically significant differences between wakefulness and sleep. Data are presented as box-and-whisker plots. The solid horizontal line indicates the median, the dashed line represents the mean, boxes denote the interquartile range (25–75th percentile), and whiskers extend to the minimum and maximum values excluding outliers. Gray circles represent wakefulness, white circles represent sleep, and black circles indicate outliers.

**Figure 3:**
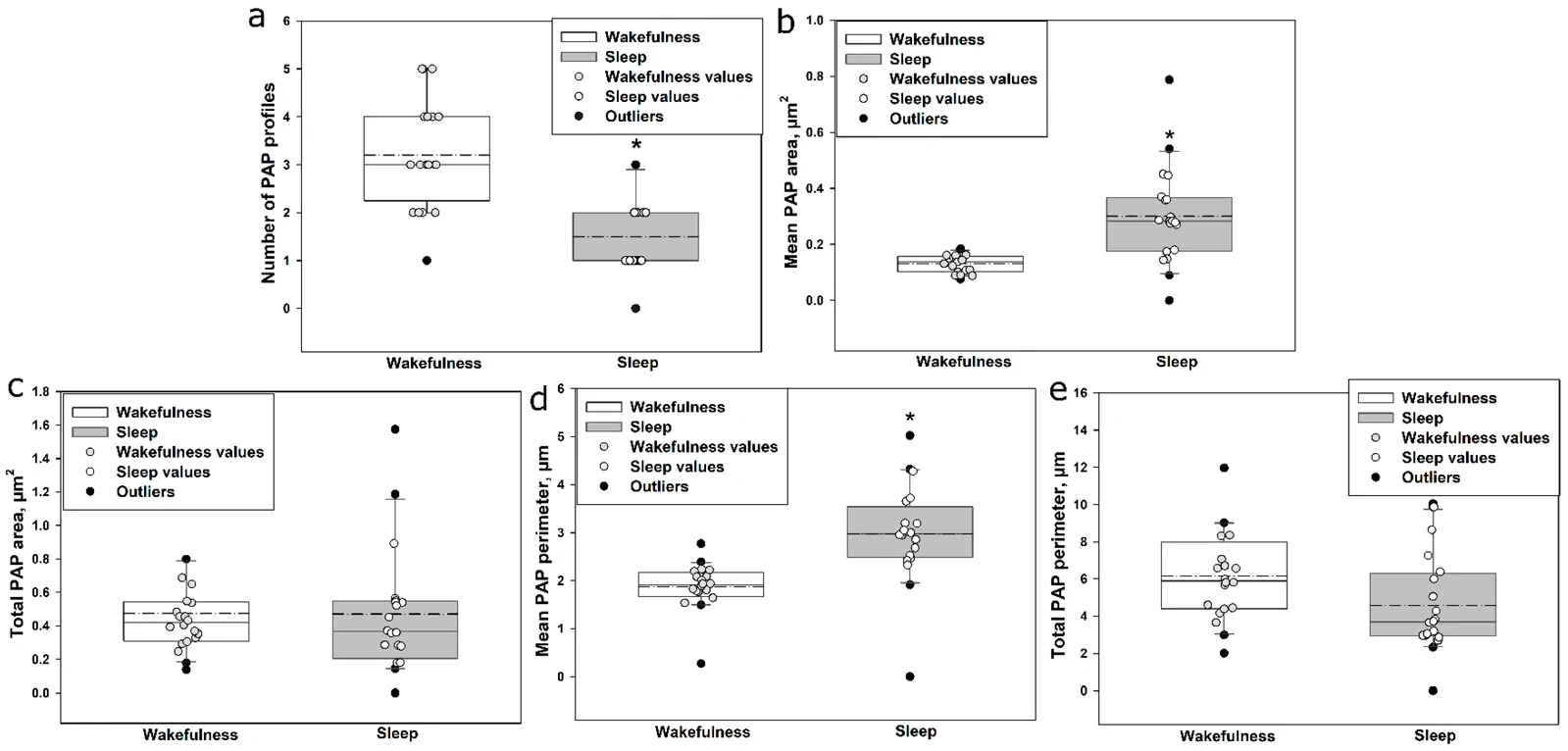
Quantitative and morphometric characteristics of perisynaptic astrocytic processes (PAPs) in the motor cortex of bats during wakefulness and sleep. (**a**) Number of PAP profiles contacting synapses. (**b**) Mean PAP area. (**c**) Total PAP area. (**d**) Mean PAP perimeter. (**e**) Total PAP perimeter. Analyses included 20 neuropil fields per physiological state, obtained from 6 animals during wakefulness and 6 animals during sleep. Statistical analysis was performed using the two-sided Mann–Whitney U test with Holm’s sequentially rejective Bonferroni procedure to control for multiple comparisons within this family of PAP endpoints. * *p* < 0.05 after correction indicates statistically significant differences between wakefulness and sleep. Data are presented as box-and-whisker plots. The solid horizontal line indicates the median, the dashed line represents the mean, boxes denote the interquartile range (25–75th percentiles), and whiskers extend to the minimum and maximum values excluding outliers. Gray circles represent wakefulness, white circles represent sleep, and black circles indicate outliers.

**Figure 4:**
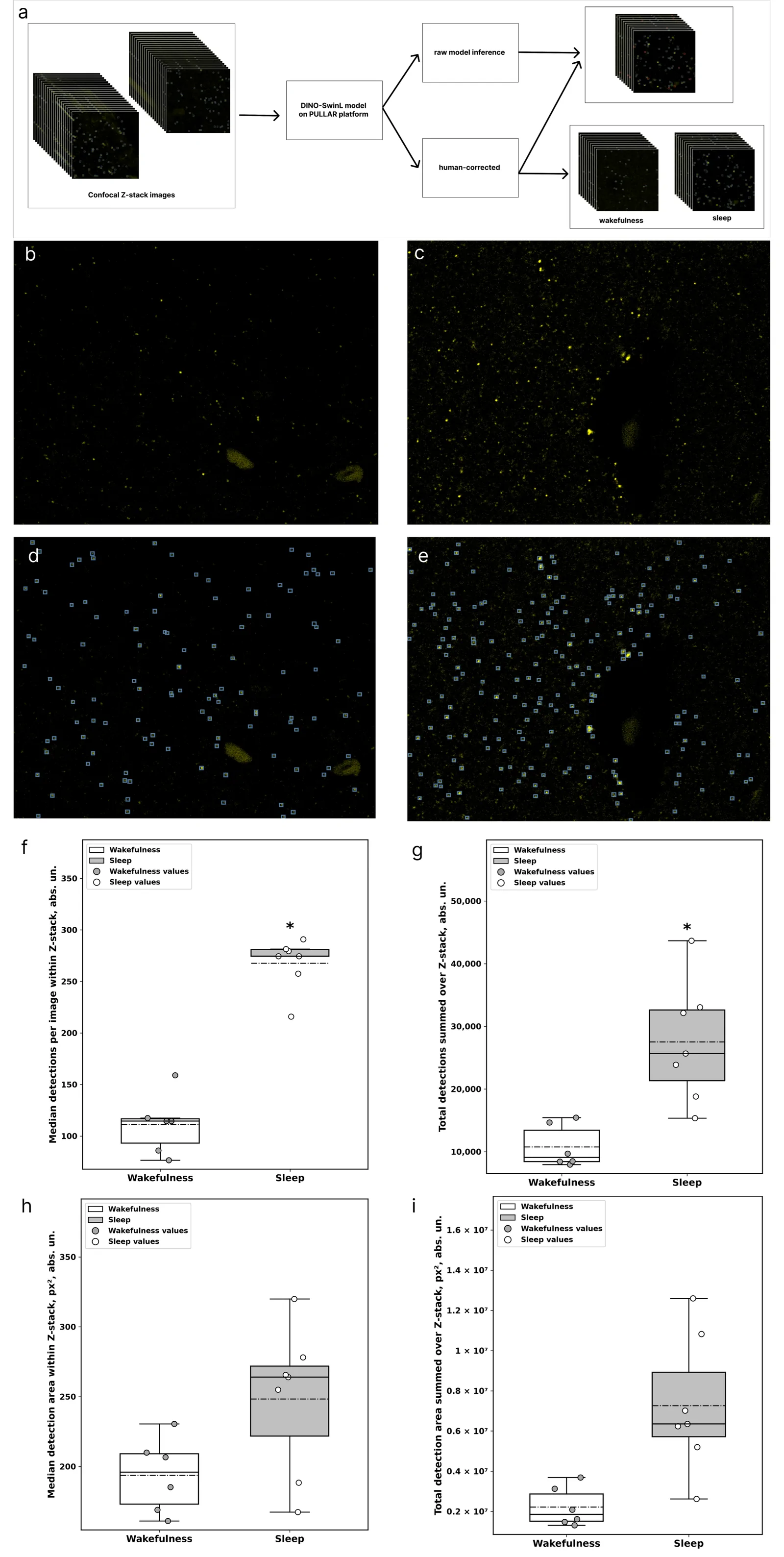
Human-corrected qualitative and quantitative analysis of Cx43 gap junctions in the bat motor cortex during wakefulness and sleep. (**a**) Overview of the Cx43 gap-junction detection and comparison pipeline. Representative confocal Z-stacks (left; individual optical sections shown as stacked panels) were processed by a DINO-SwinL object-detection model deployed on the PULLAR labeling and training platform, producing raw model inference. A subset of these detections was independently reviewed and corrected by a blinded human annotator to generate the human-corrected reference set. Raw and human-corrected detections on the same images were compared directly to assess detection-source agreement. The human-corrected detections were additionally used as the reference for comparing gap-junction counts between physiological states, wakefulness and sleep (bottom right). Panels (**b**–**e**) show representative single-plane confocal images. The left column corresponds to wakefulness and the right column to sleep. The top row presents the original Cx43-immunolabeled images, whereas the bottom row shows the corresponding images after manual correction of automated detections; blue bounding boxes indicate identified Cx43-positive gap junctions. (**b**) Original image, wakefulness. (**c**) Original image, sleep. (**d**) Human-corrected detections in the wakefulness image. (**e**) Human-corrected detections in the sleep image. Panels (**f**–**i**) present quantitative analysis performed at the Z-stack level, with each Z-stack representing one independent animal (wakefulness, *n* = 6 animals; sleep, *n* = 7 animals; 290 awake and 366 sleep images with valid human-corrected detections after filtering). (**f**) Median number of Cx43 detections per image. (**g**) Total number of Cx43 detections per Z-stack. (**h**) Median detection area. (**i**) Total detection area. Data are presented as box-and-whisker plots: the solid horizontal line within each box indicates the median, the dash-dot line indicates the mean, the box boundaries represent the 25th and 75th percentiles, and the whiskers extend to the minimum and maximum values excluding outliers. Statistical analysis was performed using the two-sided Mann-Whitney U test with Holm’s sequentially rejective Bonferroni correction across the four plaque metrics in panels (**f**–**i**). Between-group comparisons used only human-corrected detections. * *p* < 0.05 after correction indicates statistically significant differences between the wakefulness and sleep conditions. Black circles represent wakefulness, and white circles represent sleep.

**Figure 5:**
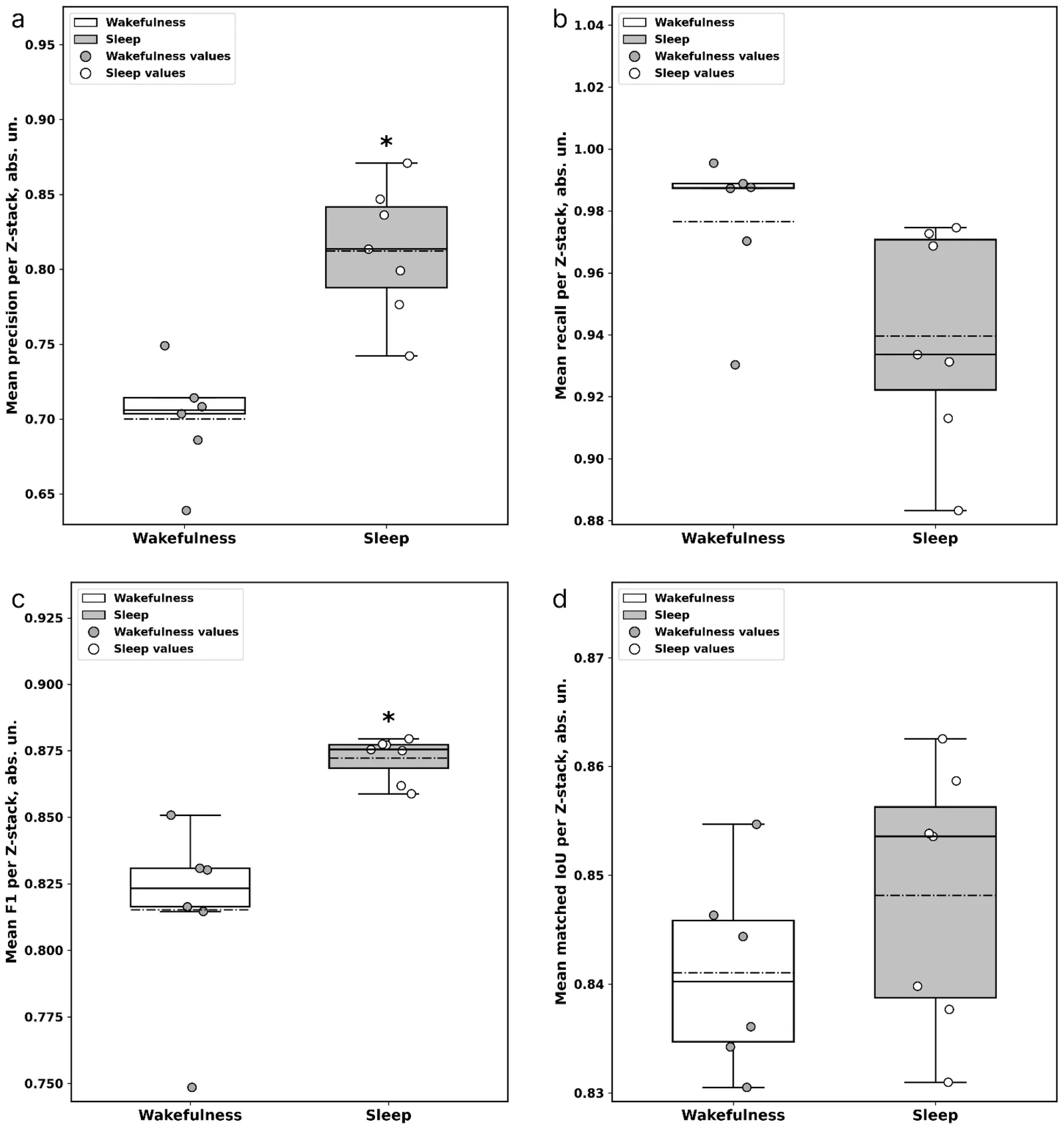
Performance of automated Cx43 gap-junction detection relative to the human-corrected reference at the Z-stack level. (**a**) Mean precision per Z-stack. Each point represents one Z- stack. (**b**) Mean recall per Z-stack. (**c**) Mean F1 score per Z-stack. (**d**) Mean matched intersection over union (IoU) per Z-stack (wakefulness, *n* = 6; sleep, *n* = 7). Data are presented as box-and-whisker plots: the solid horizontal line indicates the median, the dash-dot line indicates the mean, the box represents the interquartile range (25–75th percentile), and whiskers extend to the most extreme values within 1.5× the interquartile range. Points beyond the whiskers are shown as outliers. Statistical significance was assessed using the two-sided Mann–Whitney U test with Holm’s sequentially rejective Bonferroni correction across the four performance metrics. Significant differences were observed for precision ((**a**), *p* = 0.0023) and F1 score ((**c**), *p* = 0.0012), whereas recall ((**b**), *p* = 0.051) and mean matched IoU ((**d**), *p* = 0.295) did not differ significantly between physiological states. Asterisks indicate statistically significant differences between wakefulness and sleep after Holm correction (* *p* < 0.05).

**Figure 6:**
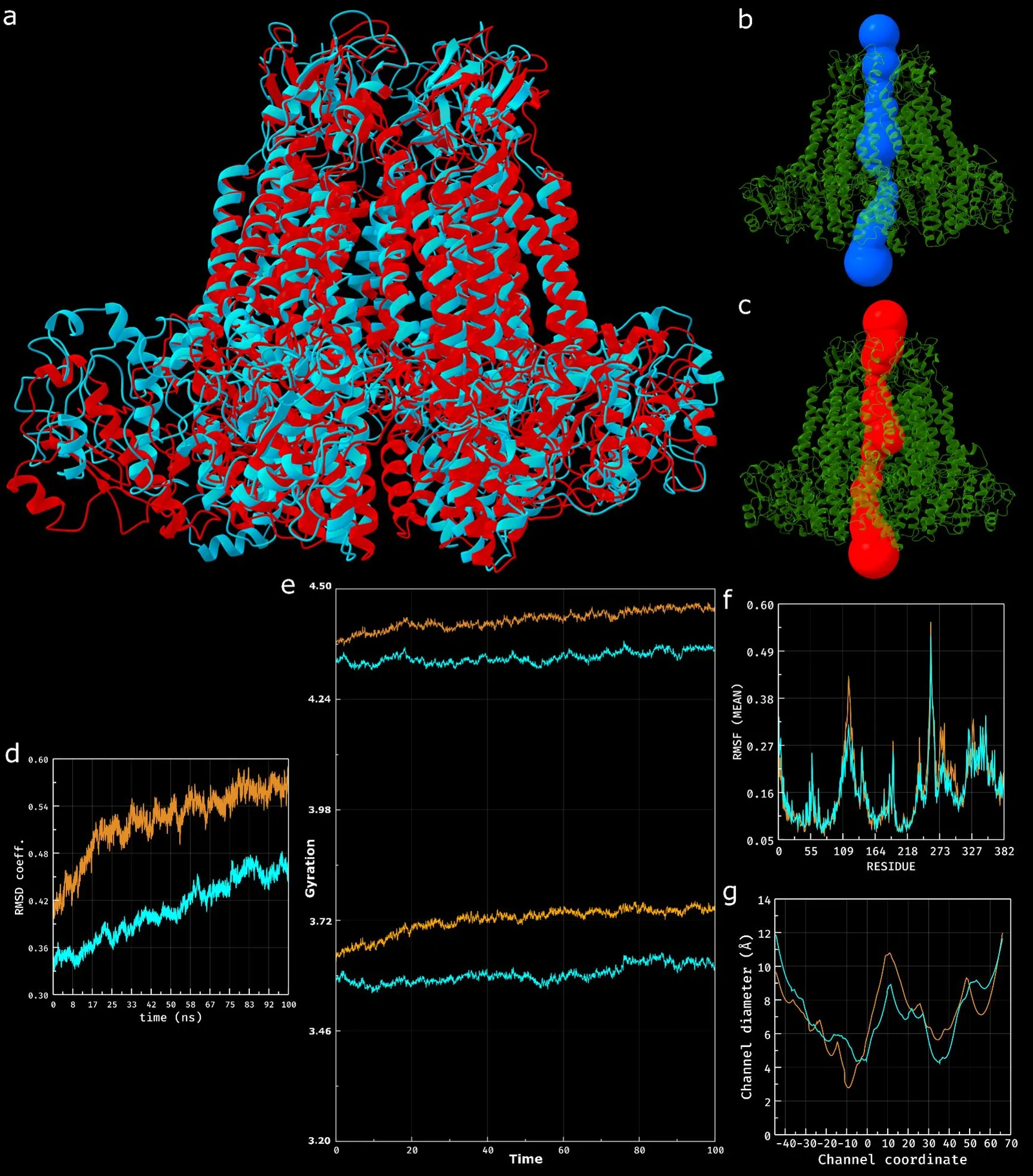
Structural alterations and quantitative analysis of the physiological and protonated Cx43 hemichannels after 100 ns of molecular dynamics simulations. (**a**) Superimposed and structurally aligned physiological (blue) and protonated (red) Cx43 hemichannel models after 100 ns of molecular dynamics simulation. The RMSD between the final structures was 2.803 Å. (**b**) Three-dimensional visualization of the pore profile in the physiological hemichannel. (**c**) Three-dimensional visualization of the pore profile in the protonated hemichannel. (**d**) Time-dependent changes in the RMSD of hemichannel atomic coordinates. (**e**) Time-dependent changes in the radius of gyration. The upper panel shows the overall radius of gyration, whereas the lower panel shows the radius of gyration along the Z-axis. (**f**) RMSF of amino acid residues in one representative subunit after hexamer alignment (average values for that subunit). (**g**) Pore diameter as a function of the channel coordinate determined using the HOLE-2 program; the origin corresponds to the center of the hemichannel model. Orange indicates the reference hemichannel, and cyan indicates the protonated form.

**Table 1:** Animal-level morphometry of the motor-cortex neuropil in *Nyctalus noctula*. Values are the median [Q1–Q3] of animal means (wakefulness, *n* = 6; sleep, *n* = 6). Groups were compared with the two-sided Mann–Whitney U test. *p* is the uncorrected test result; Holm *p* is the value after Holm’s sequentially rejective Bonferroni correction within this family of ten endpoints. The asterisk (*) marks the endpoint that remained significant after this correction (Holm *p* < 0.05). r is the rank-biserial correlation: a positive value indicates a higher result during wakefulness, and a negative value a higher result during sleep. Image-level distributions are shown in Figure 2.

Endpoint	Wakefulness	Sleep	*p*	Holm *p*	*r*
Number of synapses	6.05 [5.27–6.73]	3.63 [3.60–3.79]	0.008	0.072	0.94
Number of synaptic zones	7.98 [7.54–10.25]	4.53 [4.21–5.79]	0.016	0.105	0.86
Total active-zone length, µm	2.01 [1.91–2.40]	1.22 [1.14–1.34]	0.004	0.040 *	0.94
Mean active-zone length, µm	0.233 [0.217–0.254]	0.251 [0.231–0.264]	0.485	1	−0.28
Number of neuronal processes	11.29 [10.20–13.67]	7.28 [6.62–7.94]	0.015	0.105	0.83
Total neuronal-process area, µm^2^	5.10 [4.05–6.10]	3.12 [2.66–3.20]	0.015	0.105	0.83
Mean neuronal-process area, µm^2^	0.431 [0.389–0.457]	0.418 [0.326–0.438]	0.589	1	0.22
Number of mitochondria	10.82 [10.10–11.71]	8.35 [7.64–9.00]	0.010	0.080	0.92
Mean mitochondrial area, µm^2^	0.186 [0.144–0.211]	0.219 [0.203–0.238]	0.240	0.960	−0.44
Total mitochondrial area, µm^2^	2.10 [1.60–2.51]	1.90 [1.87–2.02]	0.818	1	0.11

**Table 2:** Animal-level morphometry of perisynaptic astrocytic processes in the motor cortex of *Nyctalus noctula*. Values are the median [Q1–Q3] of animal means (wakefulness, *n* = 6; sleep, *n* = 6). Groups were compared with the two-sided Mann–Whitney U test. *p* is the uncorrected test result; Holm *p* is the value after Holm’s sequentially rejective Bonferroni correction within this family of five endpoints. The asterisk (*) marks endpoints that remained significant after this correction (Holm *p* < 0.05). r is the rank-biserial correlation: a positive value indicates a higher result during wakefulness, and a negative value a higher result during sleep. Image-level distributions are shown in Figure 3.

Endpoint	Wakefulness	Sleep	*p*	Holm *p*	*r*
Synapses contacted by PAPs	3.12 [2.68–3.27]	1.24 [1.20–1.32]	0.008	0.024 *	0.94
Mean PAP area, µm^2^	0.121 [0.114–0.136]	0.275 [0.181–0.378]	0.002	0.010 *	−1.00
Total PAP area, µm^2^	0.381 [0.359–0.418]	0.401 [0.295–0.458]	0.937	0.937	−0.06
Mean PAP perimeter, µm	1.84 [1.78–2.02]	3.01 [2.77–3.25]	0.002	0.010 *	−1.00
Total PAP perimeter, µm	5.61 [5.37–6.11]	4.09 [3.78–4.33]	0.132	0.264	0.56

## Data Availability

All data obtained in this study are available in the GitHub repository https://github.com/rosali3/bat_article (accessed on 9 September 2026). Additional data are available from the corresponding author upon request.
